# Supplementary material for: Postpartum cytokine shifts and IL-10–mediated immune suppression in malaria-infected primigravid women
Source: PLoS One. 2026 Feb 10;21(2):e0342675. doi: 10.1371/journal.pone.0342675 (PMC12890173; doi:10.1371/journal.pone.0342675)
Supplement: S1 Fig — (DOCX) [file pone.0342675.s001.docx]

| **Malaria in**  **primigravid women** | **Postpartum**  **cytokine shifts** | **Persistent**  **parasitaemia** |
| --- | --- | --- |
| **** | **** | **** |
| **Postpartum cytokine shifts and IL-10-mediated immune suppression in malaria-infected primigravid women** | | |
