# Supplementary material for: Postpartum cytokine shifts and IL-10–mediated immune suppression in malaria-infected primigravid women
Source: PLoS One. 2026 Feb 10;21(2):e0342675. doi: 10.1371/journal.pone.0342675 (PMC12890173; doi:10.1371/journal.pone.0342675)
Supplement: Attachment — Submitted filename: PONE - Traore et al_Revision Note - Response to Reviewers.pdf [file pone.0342675.s003.pdf]

Response to reviewers

Manuscript title: *Postpartum cytokine shifts and IL-10–mediated immune suppression in malaria-infected primigravid women*

Manuscript number: PONE-D-25-29041

Dear Academic Editor and Reviewer,

We thank you sincerely for your thoughtful and constructive review of our manuscript. We have carefully addressed each point raised, and we appreciate the opportunity to improve the clarity, rigor, and contextual relevance of our work. Below is a point-by-point response to all reviewer comments and editorial requests.

Reviewer #1 Comments

Major Comments

Comment 1:

There is an abundance of published information concerning the associations between infection during pregnancy with P. falciparum and elevated plasma cytokine levels, both in peripheral and placental compartments. Much of the relevant literature is not cited by the authors. This should be rectified. Particularly pertinent to the authors' findings are those studies that have clearly identified elevated levels of IL-10 in infected mothers.

Otherwise, there is one essential piece of information that the authors have not included in their manuscript, for reasons that are not at all clear.

Author response:

We thank the reviewer for this important observation and agree that our findings should be more clearly contextualized within the existing literature.

We have revised the Discussion section to include and synthesize key studies documenting elevated IL-10 levels and broader cytokine alterations in *Plasmodium falciparum* - infected pregnant women, in both peripheral and placental compartments.

These additions better situate our results within established evidence on the immunoregulatory role of IL-10 during pregnancy-associated malaria and strengthen the interpretation of our findings.

Comment 2:

They identified infections in peripheral blood through the use of different techniques, including, most importantly, a PCR-based method. The particular method employed, relying on amplification of polymorphic regions of the MSP2 gene, would, I assume, allow for confirmation of whether or not infections detected in the post-partum period comprised newly-acquired infections or - perhaps most

likely - infections that were already present prior to delivery that had persisted post-delivery. In the latter case, I would conjecture that it would be no surprise at all to find that the (anti-inflammatory) IL-10-dominated cytokine profile observed prior to delivery in infected mothers was, as it were, carried over - persisted, along with the infection - into the post-partum period in those that failed to clear their infections at or around the time of delivery. The authors should therefore consider including the relevant PCR-derived information in the Results, with an appropriate modification of the Discussion.

Author response:

We sincerely thank the reviewer for this highly insightful comment. We agree that distinguishing whether postpartum infections were newly acquired or persistent from the antenatal period is essential for interpreting the observed cytokine dynamics, especially the sustained IL-10 production.

In response, we have added clarifications in the Methods and Discussion sections to emphasize that while our nested PCR assay targeted polymorphic regions of the *msp2* gene, it was optimized solely for sensitive detection of *Plasmodium falciparum*, not for genotyping. The protocol was not designed to differentiate parasite clones or determine infection multiplicity and persistence across time points.

We now explicitly acknowledge this limitation in the manuscript and highlight the utility of *msp2* genotyping to distinguish between new and persistent infections in longitudinal malaria studies. Incorporating such genotyping in future studies would allow a more definitive assessment of infection dynamics and their immunological correlates, including whether sustained IL-10 expression postpartum is linked to persistent parasitaemia. This would directly test the reviewer's well-argued hypothesis regarding IL-10 carry-over from unresolved infections at delivery.

These points have been added to the Discussion to strengthen the interpretation of our findings in light of this important methodological consideration.

Minor Comments

Comment 3:

*Table 1: Line 180 of Results states that the mean parasite density at delivery was 540.9, but a different number appears in Table 1. This anomaly should be corrected, and the numbers of individuals with peripheral infections identified by microscopy should also be added to the Table. An * is missing for "Parasite density of peripheral infection at delivery"; a third * is missing for "Placental malaria at delivery".*

Author response:

We thank the reviewer for pointing out this inconsistency and the missing annotations. We have addressed all points as follows:

- ***Mean parasite density at delivery*:**

  We corrected the discrepancy by updating the value in the Results section to match the value reported in Table 1, based on the microscopy-confirmed data.

- ***Microscopy-positive infections:***

  We added the number of microscopy-detected peripheral infections at delivery to Table 1, as requested.

- ***Asterisks for clarification:***

  We have now included:

  - A single asterisk (*) next to the line reporting parasite density of peripheral infection, indicating that it was determined by microscopy.
  - A third asterisk (***) for placental malaria, to match the corresponding footnote referring to diagnosis by placental histology.

All changes have been incorporated into the revised version of Table 1 and reflected in the updated manuscript.

Comment 4:

In various places throughout the manuscript (Methods, Table 5, Figure 1), the authors erroneously refer to "serum" whereas they were actually using "plasma". This should be corrected throughout.

Author response:

Thank you for pointing this out. All references to "serum" have been corrected to "plasma" throughout the manuscript, including the Methods, Table 5, Figure 1, and relevant figure legends.

PLOS ONE journal requirements

1. **Role of funders**

Author response:

We have included the following statement in our cover letter and manuscript:

"The funders had no role in study design, data collection and analysis, decision to publish, or preparation of the manuscript."

2. **Ethics statement**

Author response:

We moved the ethics statement to the Methods section under a new subheading "Ethical Approval." It reads:

The institutional review board of Centre Muraz/IRSS granted ethical approval for this study (Reference A007-2014/CEI-CM dated 12th February 2014). The approval was amended on July 25, 2024, and subsequently extended on September 29, 2015, for an additional year of follow-up for women (July 25, 2014 – July 25, 2016).

It has been removed from all other sections.

3. **Reference list**

Author response:

The reference list has been carefully reviewed and updated. No retracted papers are cited. We added several foundational references regarding cytokine responses in malaria-infected pregnant women.

We believe these changes substantially improve the manuscript and respectfully submit our revised version for your consideration. Thank you again for your helpful feedback and support.

4. **Figures updated to comply with PLOS format**

Author Response:

All figures have been revised and uploaded in TIFF format at 300 dpi resolution, in line with PLOS ONE requirements.
